# Factors associated with the quality of life of multi-professional health residentes

**DOI:** 10.1590/0034-7167-2021-0541

**Published:** 2022-06-24

**Authors:** Daiane Dal Pai, Luciana Olino, Lidiellen Eich, Raquel Lautenchleger, Marcelo Nunes da Silva Fernandes, Juliana Petri Tavares

**Affiliations:** I Universidade Federal do Rio Grande do Sul. Porto Alegre, Rio Grande do Sul, Brazil.; II Universidade Federal de Ciências da Saúde de Porto Alegre. Porto Alegre, Rio Grande do Sul, Brazil.; III Universidade Federal de Santa Maria. Santa Maria, Rio Grande do Sul, Brazil.

**Keywords:** Mental Health, Quality of Life, Internship, Nonmedical, Internship and Residency, Personal Satisfaction, Salud Mental, Calidad de Vida, Internado no Médico, Internado y Residencia, Satisfacción Personal, Saúde Mental, Qualidade de Vida, Internato não Médico, Internato e Residência, Satisfação Pessoal

## Abstract

**Objectives:**

to analyze factors associated with the quality of life of multi-professional health residents.

**Methods:**

cross-sectional and analytical design, carried out with 94 residents who answered a questionnaire containing general data, the Maslach Burnout Inventory, the Self Reporting Questionnaire, and the World Health Quality of Life. Data was submitted to multiple linear regression, considering p < 0.05.

**Results:**

minor mental disorders increased, respectively, by 0.31, 0.64 and 0.35 in the Physical, Psychological and General domains of quality of life. Emotional exhaustion had an inverse influence of 0.28 on the Physical and Environment domains. Satisfaction with residence increased the overall quality of life outcome by 0.20; Living alone, at 0.02 the Psychological domain; and Race/color, at 0.19 the Environment domain.

**Conclusions:**

there is a relationship between emotional exhaustion of the burnout, minor psychic disorders and the quality of life of multi-professional residents.

## RESUMO

**Objetivos:**

analisar fatores associados à qualidade de vida de residentes multiprofissionais em saúde.

**Métodos:**

delineamento transversal e analítico, realizado com 94 residentes que responderam a um questionário contendo dados gerais, o *Maslach Burnout Inventory*, o *Self-Reporting Questionnaire* e o *World Health Quality of Life*. Os dados foram submetidos à regressão linear múltipla, considerando *p* < 0,05.

**Resultados:**

os distúrbios psíquicos menores incrementaram, respectivamente, 0,31, 0,64 e 0,35 nos domínios Físico, Psicológico e Geral da qualidade de vida. O Desgaste emocional apresentou influência inversa de 0,28 sobre os domínios Físico e Meio ambiente. A Satisfação com a residência incrementou em 0,20 o desfecho geral da qualidade de vida; Residir sozinho, em 0,02 o domínio Psicológico; e a Raça/cor, em 0,19 o domínio Meio ambiente.

**Conclusões:**

existe relação entre Desgaste emocional do *burnout*, Distúrbios psíquicos menores e a qualidade de vida de residentes multiprofissionais.

## RESUMEN

**Objetivos:**

analizar factores relacionados a la calidad de vida de residentes multiprofesionales en salud.

**Métodos:**

delineamento transversal y analítico, realizado con 94 residentes que respondieron a una encuesta conteniendo datos generales, el *Maslach Burnout Inventory*, el *Self-Reporting Questionnaire* y el *World Health Quality of Life*. Los datos fueron sometidos a la regresión linear múltiple, considerando *p* < 0,05.

**Resultados:**

los disturbios psíquicos menores incrementaron, respectivamente, 0,31, 0,64 y 0,35 en los dominios Físico, Psicológico y General de la calidad de vida. El Desgaste emocional presentó influencia inversa de 0,28 sobre los dominios Físico y Medio ambiente. La Satisfacción con la residencia incrementó en 0,20 el desfecho general de la calidad de vida; Vivir solo, en 0,02 el dominio Psicológico; y la Raza/color, en 0,19 el dominio Medio ambiente.

**Conclusiones:**

hay relación entre Desgaste emocional recurrente del *burnout*, Disturbios psíquicos menores y la calidad de vida de residentes multiprofesionales.

## INTRODUCTION

The creation of the Multi-professional Residency program was based on Law No. 11,129 of 2005, being governed by the principles and guidelines of the Unified Health System^(1)^. It is a *lato sensu* specialization where the main focus is practical activities. This type of service education aims to promote in-service education and favor a qualified insertion of new professionals in the labor market^(2)^. The resident needs to dedicate himself exclusively to the program that provides teaching-assistance supervision, in addition to the provision of a paid scholarship^(1)^. In the work routine, there is a great demand on the residents^(3)^; and studies have pointed out that the long working hours together with the occurrence of daily tension, the inability of workers to respond to the daily challenges of the profession without organizational support and the lack of preparation of supervisors can culminate in psychological distress, fatigue, sleep difficulties and physical exhaustion of the resident. , affecting their quality of life^(3,4,5)^.

Research has shown minor psychic disorders among residents^(3,4,5,6)^, characterized by symptoms such as tiredness, memory deficit, sadness, anxiety, irritability, concentration problems, somatic complaints and feelings of worthlessness^(7)^. There are also reports of impaired sleep quality with potential impairment and development of burnout syndrome^(5,6,7,8)^, characterized by emotional exhaustion, depersonalization and decreased professional fulfillment^(9-10)^.

In order to respond to the needs of daily work, in addition to psychological conditions, some workers may resort to substance use to face the daily challenges of the profession. Studies^(11-12)^ have pointed out that work-related stress can drive individuals to consume psychoactive substances, which cause damage to health and interfere with quality of life.

The quality of life is influenced by positive and negative aspects related to the social, cultural, and environmental context. These aspects are physical (vitality, tiredness), psychological (emotions), level of independence, social relationships (support network), surroundings (accessibility to health care) and personal and spiritual beliefs (meaning of life)^(13)^. Given the above, the need for studies aimed at this group of professionals, which represents an important contribution to the Unified Health System, became evident; and the question was: What factors are associated with the quality of life of multi-professional health residents?

## OBJECTIVES

To analyze factors associated with the quality of life of multiprofessional health residents.

## METHODS

### Ethical aspects

This study was approved by the Research Ethics Committee of the institution where the study was carried out. All participants signed the Free and Informed Consent Form (ICF), in compliance with the provisions of the National Health Council Resolution No. 466/2012, which deals with research in human beings.

### Study design, period, and location

This is a cross-sectional and analytical study, guided by the STROBE tool (Strengthening the Reporting of Observational Studies in Epidemiology). Data collection took place from May to August 2019. The research setting was the *Hospital de Clínicas de Porto Alegre* (HCPA), a public university institution, academically linked to the Federal University of Rio Grande do Sul (UFRGS). The Integrated Multi-professional Health Residency (RIMS) at HCPA is made up of programs in the following areas: Primary Health Care, Critical Adults, Comprehensive Care for Adult Surgical Patients, Cardiovascular Care, Maternal and Child Care, Comprehensive Care for Drug Users, Control of Hospital Infection, Mental Health, Onco-Hematology and Child Health.

### Population, sample, inclusion, and exclusion criteria

Multi-professional HCPA residents from all RIMS programs (total of 106 residents), enrolled in 2019 and with active employment during the data collection period, from the professions of nursing, pharmacy, physiotherapy, speech therapy, nutrition, psychology, social work, physical education, and occupational therapy. The exclusion criterion was being away during the data collection period due to vacation, medical certificate, leave or disconnection from the multi-professional health residency. The sample size calculation considered the lowest correlation (0.326) between one of the associated factors (burnout) and the quality of life outcome^(14)^, statistical power of 80%, for a confidence level of 95%. The minimum sample expected was 72 residents, and the final sample of this study consisted of 94 residents.

### Study protocol

Data were collected by previously trained students from UFRGS, with the face-to-face application of a closed questionnaire, created by the researchers and composed of sociodemographic and employment data, including variables such as gender, date of birth, marital status, profession, education, who lives with and days away from work. Substance use and self-medication are questions that addressed the use of controlled psychoactive medication, use of controlled psychoactive medication before residency, addition of controlled psychoactive medication, use of over-the-counter medication, use of over-the-counter medication without professional indication, use and increased use of caffeine, in addition to the influence of caffeine consumption on sleep.

Also used were:

- World Health Quality of Life (WHOQOL-Bref) – Developed by the Quality of Life Group of the World Health Organization (WHO) and validated in Brazil^(15)^, it contains 26 questions, two of which are general (Global Quality of Life and Health Perceptions) general), and the other 24 comprise four domains: Physical (pain, energy, sleep, mobility, activities of daily living, medication/treatments, ability to work); Psychological (positive/negative feelings, self-esteem, body image, spirituality); Social relationships (personal relationships, social support and sexual activity); Environment (physical security, home environment, financial resources, health and social care, opportunities to acquire information and skills, recreation/leisure, physical environment and transportation)^(16).^
- Self-Report Questionnaire (SRQ-20) – Enables tracking of minor psychiatric disorders; was developed by Harding in 1980, funded by the WHO, validated in Brazil^(17)^ and has 20 “yes” and “no” choice questions. The instrument has 80% specificity and 83% sensitivity for detecting MPD cases compared to the standard psychiatric interview using the *semi-structured Clinical Interview Schedule* ^(17)^. The cut-off point used for detecting MPD were values equal to or greater than seven positive responses.
- Maslach Burnout Inventory - MBI (burnout) - It was developed in 1978 by Christina Maslach and Susan Jackson and validated in Brazil^(18)^. It is an instrument composed of a Likert-type scale ranging from 0 to 6, capable of detecting the *burnout* syndrome and the factors that compose it: emotional exhaustion, depersonalization, and professional fulfillment.

### Analysis of results and statistics

Data were entered into a Microsoft Excel spreadsheet, analyzed using the Statistical Package for the Social Sciences, version 20 (SPSS® Statistics); and submitted to descriptive and inferential statistics. Qualitative variables were described using relative and absolute frequencies. Numerical variables were presented through measures of central tendency (mean or median) and dispersion (standard deviation or interquartile ranges), according to the Shapiro-Wilk test result, asymmetry, and kurtosis values. The analysis of association between the factors under study (MPD and burnout syndrome) and the outcome (quality of life) were performed using Tukey’s test, Student’s t test and Pearson’s correlation. In the association with medication use, Student’s t tests (variables with symmetrical distribution) and Mann-Whitney (variables with asymmetric distribution) were used. Values of p < 0.05 were considered significant. Multiple linear regression using the Stepwise method was performed to select the regression model for variables that were associated with the outcome (quality of life) with a confidence level of 95% (p ≤ 0.05). The variables with univariate analyzes of p < 0.20 were included in the model; and data with a two-tailed p lower than 0.05, or with a confidence interval of 95%, were considered as statistically significant differences.

## RESULTS

Most residents were female (n = 84; 89.4%), with a median age of 26 years (22-53), self-reporting as white (n = 78; 83%), living with the family (n = 31; 36%), without a partner (n = 63; 67.7%), with an average of 17.2 years of schooling (SD± 2.0). 17 nutritionists (18.1%), 16 psychologists (17%), 14 social workers (14.9%), 14 nurses (14.9%), 10 physiotherapists (10.6%) participated., 8 pharmacists (8.5%), 8 physical educators (8.5%), 5 speech therapists (5.3%) and 2 occupational therapists (2.1%). 49 professionals (52.7%) were in their first year of residency; and 44 in the second year (47.3%).

The quality-of-life domains presented means above 55, with 59.57 (± 13.14) being the mean of the Physical domain; 55.50 (± 13.97) in the Psychological domain, 60.10 (± 17.70) in the Social relationships, 56.74 (± 13.66) in the environment, and the average of the General domain of quality of life was 55.45 (± 19.18).

Table 1 describes the association of psychological factors (burnout and minor mental disorders) with the residents’ quality of life.

**Table 1:** Association between quality of life and the dimensions of burnout and Minor psychological disorders among multi-professional health residents

Variables	Physical	Psychological	Quality of life Social	Environmental	General
Emotional exhaustion					
Low	67.85(±11.37)^a^	62.78(±16.51)^a^	66.95(±19.09)^a^	63.36(±12.74)^a^	64.65(±19.49)^a^
Moderate	58.66(±11.23)^b^	53.98(±11.97)^b^	60.10(±15.72)^a^	56.25(±12.44)^a^	52.92(±18.46)^a^ ^b^
High	48.61(±12.01)^c^	48.61(±12.01)^b^	49.07(±15.36)^b^	47.39(±12.91)^b^	47.22(±15.19)^b^
*p* < 0.05 General	**0.000**	**0.001**	0.003	**0.000**	0.004
Professional achievement					
Low	65.17(±14.48)^a^	57.98(±17.50)^a^	69.09(±14.63)^a^	62.23(±11.68)^a^	59.89(±19.84)^a^
Moderate	59.24(±11.29)^ab^	55.22(±12.79)^a^	57.67(±17.22)^b^	54.84(±12.34)^a^	52.20(±19.47)^a^
High	53.38(±13.70)^b^	53.28(±12.23)^a^	55.26(±19.28)^b^	54.93(±17.68)^a^	58.55(±16.69)^a^
*p* < 0.05 General	0.012	0.540	**0.012**	0.073	0.199
Depersonalization					
Low	58.51(±13.44)^a^	53.04(±16.13)^a^	63.46(±19.73)^a^	54.92(±14.76)^a^	52.40(±20.92)^a^
Moderate	60.15(±12.27)^a^	60.15(±12.27)^a^	59.07(±15.67)^a^	58.05(±11.56)^a^	54.72(±19.27)^a^
High	59.62(±14.89)^a^	59.62(±14.89)^a^	58.33(±19.29)^a^	56.25(±16.31)^a^	60.32(±16.70)^a^
*p* < 0.05 General	0.881	0.545	0.522	0.641	0.336
Dimensions of burnout					
Emotional exhaustion	r = -0.51**	r = -0.42**	r = -0.38**	r = -0.42**	r = - 0.36**
Professional achievement	r = -0.32*	r = -0.18	r = -0.26*	r = -0.19	r = -0.47
Depersonalization	r = 0.24	r = 0.48	r = -0.11	r = -0.01	r = 0.12
Minor psychiatric disorders					
No	70.73 (±10.64)	71.18 (±8.93)	69.44 (±19.29)	64.06 (±12.53)	68.75 (±17.67)
Yes	55.81 (±11.79)	50.17 (±11.06)	56.9 (±16.04)	54.24 (±13.20)	50.89 (±17.59)
*p* < 0.05 General	0.000	0.000	0.002	0.002	0.000
Minor psychiatric disorders	r = - 0.54**	r = -0.76**	r = -0.36**	r = -0.41**	r = -0.48**

a, b, c – Means followed by the same letter have no significant difference (p < 0.05) between the classes of each burnout domain according to the Tukey test; * p < 0.05 and **p < 0.001 in the coefficient Pearson's correlation.

Table 2 shows the correlation of variables on the consumption of medication, psychoactive substances, and self-medication with quality of life.

**Table 2:** Correlation between variables (consumption of medication, psychoactive substances, and self-medication) and quality-of-life domains

Variables	Physical	Psychological	Quality of life Social	Environmental	General
Use of caffeine					
Yes	60 (±12.3)^a^	56.3 (±13.7)^b^	60 (±17.7)^a^	57.3 (±13.0)^a^	56.4(±19.2)^b^
No	52.9 (±22.8)	44.4 (±14.1)	51.4 (±16.8)	48.9 (±21.2)	41.6 (±12.9)
*p* value	0.487	0.04	0.214	0.15	0.037
Increased caffeine consumption					
Yes	59.5 (±11.6)^a^	56.5 (±12.6)^a^	59 (±15.7)^a^	55.9 (±12.3)^a^	56 (±18.1)^a^
No	60 (±17.0)	52.8 (±17.2)	63 (±22.3)	58.9 (±17)	52 (±21.8)
*p* value	0.851	0.334	0.343	0.441	0.342
Caffeine consumption interferes with sleep					
Yes	54.4 (±10.3)^b^	51.4 (±11.4)^a^	53 (±15.9)^b^	52 (±14.9)^b^	48.8(±18.9)^b^
No	61.8 (±12.4)	57.8 (±14.0)	62.8 (±17.8)	58.9 (±12.0)	58.7 (±18.8)
*p* value	0.016	0.06	0.045	0.035	0.038
Controlled psychoactive medication use					
Yes	54.3 (±12.0)*	50.6 (±15.1)^b^	57 (±17.7)^a^	53.6 (±13.1)^a^	51 (±17.8)^a^
No	62.3 (±12.9)	58.0 (±12.7)	61.5 (±17.7)	58.4 (±13.7)	57.7 (±19.7)
*p* value	0.005	0.014	0.270	0.11	0.121
Use of controlled psychoactive medication before residency					
Yes	54.8 (±11.0)^a^	50.2 (±11.9)^a^	52 (±15.5)^b^	51.5 (±14.4)^a^	51 (±18.7)^a^
No	60.6 (±13.4)	56.7 (±14.2)	61.8 (±17.8)	57.9 (±13.3)	56.3 (±19.3)
*p* value	0.101	0.084	0.048	0.078	0.347
Addition of controlled psychoactive medication					
Yes	55.1 (±11.5)^a^	46.9 (±14.8)^b^	55 (±17.7)^a^	51.9 (±15.6)^a^	48.4 (±18)^a^
No	60.5 (±13.3)	57.3 (±13.2)	61.2 (±17.6)	57.7 (±13.1)	56.9 (±19.1)
*p* value	0.139	0.006	0.180	0.124	0.109
Use of over-the-counter medication					
Yes	58.1 (±12.3)^a^	54.6 (±13.8)^a^	60 (±19.0)^a^	54.6 (±13.6)^a^	54 (±17.9)^a^
No	61.6 (±14.1)	56.9 (±14.2)	60.1 (±15.7)	59.8 (±13.3)	56.6 (±21.1)
*p* value	0.208	0.438	0.993	0.068	0.652
Use of over-the-counter medication without professional indication					
Yes	59.3 (±16.1)^a^	60 (±12.6)^a^	63 (±16.7)^a^	52.5 (±13.4)^a^	56 (±19.7)^a^
No	57.7 (±11.2)	53.8 (±13.8)	59.9 (±19.3)	54.8 (±13.4)	54.4 (±17.4)
*p* value	0.703	0.197	0.604	0.621	0.769

^b^ p < 0,05; ^a^ p > 0,05.

Table 3 shows the results of the linear regression analysis according to the variables associated with the domains of quality of life; and these variables compose the final analysis model for this outcome.

**Table 3:** Multiple linear regression of variables associated with quality-of-life domains

Quality of life domains	Standardized beta	β (CI95%)	*p*	R²
Physical domain				
Emotional exhaustion	-0.282	-0.537(-0.996;-0.079)	0.022	0.416
Depersonalization	0.064	0.286(-0.611;1.182)	0.528	
Professional achievement	-0.047	-0.114(-0.699;0.472)	0.700	
Minor psychic disorders	-0.315	-1.058(-1.768;-0.349)	0.004	
Use of psychoactive medication	-0.099	-2.736(-7.872;2.399)	0.292	
Caffeine disrupts sleep	0.024	0.759(-5.084;6.603)	0.797	
Days away from work	-0.058	-0.059(-0.237;0.120)	0.516	
Satisfaction with the residence	0.056	1.061(-2.433;4.555)	0.547	
Marital status	0.156	4.390(0.800;9.580)	0.096	
Psychological domain				
Emotional exhaustion	-0.107	-0215(-0.599;0.168)	0.267	0.655
Depersonalization	-0.013	-0.060(-0.780;0.660)	0.869	
Professional achievement	0.101	0.261(-0.218;0.739)	0.281	
Minor psychic disorders	-0.646	-2.306(-2.908;-1.704)	**<0.001**	
Use of psychoactive medication	0.027	0.798(-4.770;6.367)	0.776	
Use of caffeine	0.126	7.450(-1.005;15.906)	0.083	
Addition of psychoactive	-0.159	-5.817(-12.391;0.757)	0.082	
Satisfaction with the residence	0.055	1.139(-1.906;4.184)	0.459	
Marital status	0.106	3.136(-1.241;7.513)	0.158	
Resides alone	-0.181	-7.515(-13.848;-1.181)	0.021	
Social relations d.				
Emotional exhaustion	-0.188	-0.481(-1.175;0.213)	0.172	0.203
Depersonalization	-0.054	-0.323(-1.591;0.945)	0.614	
Professional achievement	-0.035	-0.111(-0.963;0.740)	0.796	
Minor psychic disorders	-0.210	-0.962(-2.019;0.096)	0.074	
Prior use of psychoactive	-0.096	-4.398(-13.550;4.753)	0.342	
Satisfaction with the residence	0.067	1.757(-3.575;7.088)	0.514	
Caffeine disrupts sleep	-0.041	-1.713(-10.483;7.057)	0.699	
Environment d.				
Emotional exhaustion	-0.286	-0.561(-1.058;-0.063)	0.028	0.311
Depersonalization	0.062	0.280(-0.647;1.207)	0.550	
Professional achievement	0.038	0.095(-0.516;0.705)	0.758	
Minor psychic disorders	-0.171	-0.594(-1.377;0.190)	0.136	
Race/color	0.191	5.083(0.068;10.099)	0.047	
Satisfaction with the residence	0.182	3.608(-0.240;7.456)	0.066	
Marital status	0.163	4.675(-0.682;10.033)	0.086	
Caffeine disrupts sleep	-0.026	-0.845(-7.145;5.456)	0.790	
General domain of quality of life				
Emotional exhaustion	-0.232	-0.642(-1.328;0.044)	0.066	0.354
Depersonalization	0.71	0.461(-0.879;1.801)	0.495	
Professional achievement	0.205	0.717(-0.162;1.596)	0.108	
Minor psychic disorders	-0.354	-1.742(-2.774;-0.710)	**0.001**	
Race/color	-0.116	-0.171(-0.435;0.093)	0.201	
Satisfaction with the residence	0.196	5.492(0.297;10.688)	0.039	
Marital status	0.136	10.450(-3.333;24.232)	0.135	
Caffeine disrupts sleep	-0.089	-4.109(-12.794;4.576)	0.349	

β - regression slope; R² - coefficient of determination

It is identified that Emotional exhaustion (EE) of the burnout and Minor Psychiatric Disorders (MPD) are the variables with the greatest inverse influence on the Physical domain (PD) of quality of life. Being exposed to EE and Minor Psychic Disorders increased the outcome by 0.28 and 0.31, respectively. Both variables explain 41.6% of the FD variability. In the Psychological domain (PD), the variables MPD and Reside alone increased the outcome by 0.64 and 0.18, showing an inverse relationship and explaining the variability of this outcome in 65.5%. The study variables did not explain the variability of the Social Relations domain. The variable DE and Skin color had an inverse (-0.28) and direct (0.19) influence on the Environment domain and explained 31.1% of this outcome. The variability of the General domain of quality of life was explained in 35.4% by the variables MPD and Satisfaction with the residence: the first presented an inverse relationship and increased the outcome by 0.35; and the second, direct ratio and increased by 0.19.

## DISCUSSION

In this study, the average quality of life scores were low in all domains, being lower in the Psychological domain, followed by the Environment, Physical, and the least affected domain was Social Relations. A national study obtained similar results, indicating negative repercussions over the years of residency due to lesser commitment to social relationships^(3)^. The lowest scores in the domain of Social Relations of Quality of Life can be mitigated by the bonds that are built in the coexistence of residents, allowing for support, and understanding, since they are going through similar professional phases. A similar condition was observed in a study developed with multidisciplinary residents, in which the psychological domain seems to have been the least affected due to the routine sustained by a shared space with other residents^(19-20)^.

Furthermore, other studies also pointed to a progressive worsening in the quality of life regarding physical aspects, pain, vitality and mental health of residents^(3,21)^. Thus, the results found in this study related to the reduction in quality of life can be explained by the intense working hours, increased responsibilities, number of theoretical-practical activities and lack of time for completing the residency work. The greater commitment in the Psychological domain may be associated with the lack of time to deal with their own problems and feelings, the high demand for their own professional results, work relationships and expectations for the future.

In the present study, an inversely proportional association was observed between emotional exhaustion of the burnout and all domains of quality of life. In this regard, it is worth reflecting on the dichotomous process of being a worker and an apprentice, linked to the adaptations and challenges that the resident needs to face in their daily practices, in addition to the uncertainties about the future: all this tends to cause the physical and emotional exhaustion of these professionals. Such data corroborate the study that found a negative correlation between the Vitality variable (quality of life) and the burnout domain of Emotional exhaustion^(21)^.

Regarding professional fulfillment, it was shown to be negatively associated with the Physical and Social domains of quality of life. A Brazilian study found a positive correlation for the Decreased Feeling of Professional and Personal Achievement at Work dimension in the first year of residency^(21)^.

The use of caffeine was positively related to the average score of quality of life in the Psychological domain and was able to interfere with sleep in the Physical, Social and Environmental domains in residents who used it. Residents probably have the habit and pleasure of using caffeine due to its stimulant properties and as an aid to increase concentration, which can help with studies and keep them awake for longer, in addition to the social practice of consuming it in the form of drink (coffee). On the other hand, when consuming it in excess or at night, it can cause insomnia, irritability, agitation, tachycardia and increased anxiety^(22)^. A literature review points out that most university students find pleasure and satisfaction with the use of caffeine, but consumption should be moderate, given its influence on sleep disorders and the quality of life of students^(23)^.

As for the data on the consumption of psychoactive drugs, a significant difference can be seen between the Physical and Psychological domains; and the group that uses medication has worse scores for quality of life. In this regard, medications have been used as a faster method to cure anguish, sadness and psychological suffering, factors that contribute to the abusive increase in the use of psychotropic drugs^(24-25)^. However, this reality is not exclusive to residents, as a study with university professors identified that most professors also use medication to help cope with work-related adversities^(26)^.

When analyzing the use of controlled psychoactive drugs before residency, the group that reported this practice had worse quality of life scores compared to the group that did not use them, with a significant difference in the Social Relations domain. Regarding the addition of another controlled psychoactive medication, there was a significant difference in the Psychological domain, with worse scores. Residents often end up resorting to drug therapies to relieve symptoms caused by work overload. In the work environment of a hospital, residents assume responsibility for patient care, have extensive working hours, essential search for knowledge, demands and challenges intrinsic to the profession, which are risk factors for psychosocial illness that culminate in physical and emotional fatigue. psychological^(27)^. Therefore, it is up to the institution to consider the social context in which the professional lives and how their mental health can be affected. Thus, they resort to self-medication for the treatment of health problems, masking the symptoms and, sometimes, worsening their clinical condition. However, it is worth mentioning that the option for self-medication aims to minimize the physical or psychological disorder presented, without ensuring cure^(28)^.

Furthermore, the variable Emotional exhaustion of the burnout resulted in lower levels of quality of life in the Physical and Environment domains. Studies with workers, such as dentists^(29)^, nursing professionals^(30)^, postgraduate nursing professors^(31)^, have also shown that burnout negatively interferes with quality of life, pointing out that this is a worrying problem that needs to be addressed. be reviewed in organizations globally. Regarding residents, this result can be explained by the extensive workload and many demands assigned. In this way, time for other activities is scarce, such as leisure and even rest itself, which can generate physical and emotional exhaustion. A study revealed the excess of activities, the long workday and the scarce time for individual tasks at home as factors of dissatisfaction, culminating in emotional exhaustion^(32)^.

Regarding minor psychiatric disorders, the Physical, Psychological and Environmental domains of quality of life increased, reinforcing the interference of mental health in the quality of life of individuals. The data confirm the literature, which highlights the characteristic symptoms of MPD as a threat to functionality at work, affecting the performance and income of professionals and culminating in damage to self-esteem and quality of life^(33)^. A similar study showed a lower quality of life among individuals with MPD, in the Environmental, Psychological, Physical and Social Relations domains^(3)^. The relationship between depressive symptoms and the domains of quality of life was also found in Nursing students^(34)^, highlighting that psychic changes are present since graduation and require early detection and interventions.

In addition to psychological distress, the fact of living alone has a direct impact on the residents’ quality of life, in the Psychological domain. This fact is probably associated with the lack of emotional and financial support and also the accumulation of household chores, added to the inherent requirements of carrying out the residency. Added to this, living far from family members can influence the quality of life, due to the changes faced due to this new phase, in addition to the emergence of feelings of homesickness and sadness^(3)^. The data from this research confirm the findings of a study conducted with students in the health area, in which those who lived alone, compared to those who lived with other individuals, had lower quality of life scores in the Physical domain^(35)^.

An important factor that can directly contribute to the quality of life of residents is satisfaction with the residence. Professionals spend long hours in the work environment, where there is physical and mental exhaustion, since, in addition to working, they are learning. A study^(20)^ showed that the most interfering facet in the quality of life of multi-professional residents was job satisfaction. They develop long journeys in health care, exposed to stressful and often unfamiliar environments, which can negatively affect their performance in addition to physical and mental health. Such data converge with the findings of this study, in which satisfaction with the residence positively impacted the General domain of quality of life.

### Study limitations

A limiting factor in this study was the cross-sectional design, in which data collection takes place in a single moment, not allowing causal inference to be made.

### Contributions to the area of Nursing, Health, or Public Policy

This study contributes to the feasibility of strategies focused on the residents’ quality of life. The associated variables must be considered within the scope of the institution and public policies as important factors in the implementation of measures that promote quality of life and health of residents. In this way, it will be possible to impact patient care and reduce physical and mental illness.

## CONCLUSIONS

This study contributes to the feasibility of strategies focused on the residents’ quality of life. The associated variables must be considered within the scope of the institution and public policies as important factors in the implementation of measures that promote quality of life and health of residents. In this way, it will be possible to impact patient care and reduce physical and mental illness.

In view of the results found, it is important to point out that the residents are in the process of psychological distress, which was inversely correlated with quality of life. It is suggested to carry out studies that make it possible to intervene through strategies to minimize the effects of psychic demands on the quality of life of residents.
